# Effects of Management and Climatic Variability on Indicator Species and Biomass Production in Carpathian Mountain Grasslands

**DOI:** 10.3390/plants15020269

**Published:** 2026-01-15

**Authors:** Ioana Ghețe, Borlea Mihaela, Claudiu Șerban, Alexandru Ghețe

**Affiliations:** 1 Department of Grasslands and Forage Crops, Faculty of Agriculture, University of Agricultural Sciences and Veterinary Medicine Cluj-Napoca, Calea Mănăstur 3-5, 400372 Cluj-Napoca, Romania; ioana.ghete@usamvcluj.ro (I.G.); mihaela.achim@student.usamvcluj.ro (B.M.); 2 Research and Development Institute for Montanology, 557085 Cristian, Romania; serban.claudiu.petronius@gmail.com; 3 Department of Technical and Soil Sciences, Faculty of Agriculture, University of Agricultural Sciences and Veterinary Medicine Cluj-Napoca, Calea Mănăstur 3-5, 400372 Cluj-Napoca, Romania

**Keywords:** climate change adaptation, dry matter yield, fertilization gradients, indicator species, high-nature-value grasslands, vegetation homogenization

## Abstract

Carpathian mountain grasslands are increasingly affected by management intensification and climatic variability, with consequences for species composition and ecosystem functioning. This study assessed the long-term effects of a mineral fertilization gradient and interannual climatic variability on indicator species dynamics and biomass production in a semi-natural high-nature-value (HNV) grassland in the Apuseni Mountains, based on a 17-year field experiment. Increasing fertilization intensity promoted a clear shift from species-rich oligotrophic communities toward simplified mesotrophic and eutrophic grassland types, accompanied by a decline in indicator species richness and the increasing dominance of competitive grasses. Biomass production increased consistently along the fertilization gradient. Climate-driven effects were assessed using unfertilized control plots, allowing management effects to be disentangled from interannual climatic variability. Variations in temperature and precipitation influenced floristic composition and productivity across the years, highlighting the sensitivity of mountain grasslands to short-term climatic fluctuations. Multivariate analyses revealed increasing vegetation homogenization under high fertilization and distinct year-to-year shifts in species composition under unfertilized conditions. These results emphasize the vulnerability of Carpathian HNV grasslands to both nutrient enrichment and climatic variability, and underline the need for climate-adaptive, biodiversity-oriented management strategies.

## 1. Introduction

Grasslands are among the most extensive and valuable terrestrial ecosystems, covering approximately 40% of the continental surface and providing essential ecosystem services such as biomass production, biodiversity conservation, and carbon storage. Previous synthesis studies have shown that nutrient enrichment in semi-natural grasslands leads to a decline in species richness, a shift toward a dominance of competitive grasses, and an increase in aboveground biomass, particularly under long-term fertilization regimes [1,2,3,4]. In mountain regions, grassland ecosystems are characterized by high floristic diversity, which is maintained under low-intensity management, and by rapid vegetation responses to environmental change. These responses reflect the sensitivity of species-rich communities to variations in management practices and climatic conditions, which can quickly alter species composition and dominance patterns [5,6,7,8]. Beyond their conservation value, natural grasslands play a key role in hydrological regulation, soil protection against erosion, and the maintenance of biogeochemical cycles. Through these functions, they substantially contribute to landscape stability in mountainous and hilly regions, particularly under conditions of low-intensity land use [9,10,11,12]. In Central and Eastern Europe, semi-natural grasslands are characterized by high plant diversity, reflecting their long history of extensive management and limited nutrient input [13,14,15]. This high level of plant diversity plays a crucial role in species conservation and in maintaining long-term ecosystem functionality [16,17]. In an agroecological context, semi-natural grasslands support low-input agricultural systems by providing high-quality fodder, while also contributing to important cultural and recreational services for local communities [18,19,20]. Extensively managed grasslands generally exhibit high ecological resilience, as their vegetation structure can recover after moderate disturbances due to species-rich communities and functional redundancy [21,22]. A particularly important category at the European level is high-nature-value (HNV) grasslands, which, through traditional practices and moderate use of resources, maintain high floristic diversity and stable ecological processes [23,24,25]. These systems, often characterized by a mosaic organization of habitats, are essential refuges for many sensitive species and support the connectivity of agricultural landscapes, playing a key role in conserving genetic resources, carbon storage, and maintaining pollinator populations [26,27]. This region, which is part of the Carpathian arc, is characterized by its distinct high-altitude climate, diverse topography, and traditional land-use practices, making its grasslands particularly sensitive to environmental changes. Climate change currently represents one of the major challenges for mountain grassland ecosystems. Rising temperatures, increasing variability in precipitation, and a higher frequency of extreme weather events directly affect plant phenology, growing season length, biomass accumulation, and competitive interactions among dominant species [28,29,30]. In mountain areas, where strong microclimatic gradients are shaped by altitude, slope orientation, and local relief, these climatic changes can trigger rapid shifts in vegetation structure and species composition [31,32]. Temperatures during the early growing season (March–May) play a key role in the regeneration and early development of perennial species in mountain grasslands [33]. Warmer conditions favor species with higher thermal requirements and faster growth rates, while elevated summer temperatures increase water stress, reduce the competitiveness of mesophilic species, and promote drought-tolerant plants [34,35]. Rainfall during the critical April–June period is a key driver of grassland productivity [36,37]. Variations in water availability during this phenologically sensitive phase affect plant growth, biomass accumulation, and competitive interactions, while also influencing floristic composition by favoring species that are tolerant to changes in water and energy availability. These species, commonly referred to as indicator species, respond rapidly to environmental variation and show strong correlations with climatic factors, making them valuable tools for assessing ecosystem functioning [38,39,40]. In mountain grasslands, mesotrophic, oligotrophic, and drought-adapted species can rapidly change their abundance in response to short-term variations in climatic conditions and management intensity [41]. The rapid responses of plant communities reflect the high sensitivity of these ecosystems compared to forest ecosystems, where changes are much slower [42]. Disentangling management-driven effects from climate-driven responses remains a major challenge in mountain grassland studies, as both drivers often act simultaneously and influence vegetation structure and composition.

The importance of indicator species is significant and complex. Some of them respond mainly to management practices—fertilization, mowing, and grazing—becoming indicators of land use intensity [24,43,44,45], while others mainly react to interannual weather conditions and are used to identify the direction of climate change. Simultaneous analysis of these two categories is essential to separate the effects of management from those generated by climate variability.

Interpreting vegetation change becomes challenging when climate and management drivers act simultaneously on mountain grasslands. Management interventions such as fertilization, mowing, and grazing can amplify, dampen, or mask climate-driven signals in vegetation structure and composition [46,47]. Among these, mineral fertilization is a particularly strong driver, promoting nitrophilous species and reducing floristic diversity—an effect widely documented in HNV grasslands [15,48,49] and confirmed in recent European studies. Under such conditions, shifts in indicator species and canopy structure cannot be confidently attributed to climatic variability alone.

To distinguish climate-driven responses from management-driven effects, control variants (plots without fertilization, mowing, or grazing) provide an essential baseline. In the absence of management interventions, changes in floristic composition and biomass production can be linked with higher confidence to interannual variations in temperature and precipitation [50]. Consistent with this approach, recent studies show that mountain grasslands respond rapidly to interannual climate variability, even over short periods of 2–3 years, supporting their role as sensitive ecosystem “sensors” of climate change [37,51,52].

In this context, the present study aimed to assess the long-term effects of mineral fertilization and interannual climatic variability on mountain grasslands, based on a 17-year experiment. In the first stage, we evaluated how a fertilization gradient influenced floristic composition, indicator species, and biomass production in a high-nature-value grassland in the Apuseni Mountains. This long monitoring period provides a robust basis for interpreting vegetation dynamics and associated changes in productivity. To disentangle management-driven and climate-driven responses, unfertilized control plots were used as a baseline. In the second stage, we examined whether indicator species and biomass production varied under unfertilized conditions in response to interannual temperature and precipitation variability. This climate-focused analysis, conducted on control plots between 2015 and 2017, allowed vegetation and productivity changes to be attributed with high confidence to climatic variability alone.

Therefore, this 17-year study aimed to disentangle the combined and individual effects of management and climate variability on Carpathian mountain grasslands. Specifically, our objectives were to perform the following:

- Evaluate the long-term effects of a mineral fertilization gradient on floristic composition, indicator species, and biomass production in a high-nature-value grassland;
- Identify the indicator species associated with different fertilization levels;
- Assess the influence of interannual temperature and precipitation variability on indicator species and biomass production in the absence of management interventions;
- Determine if the indicator species identified in the fertilization experiment also respond to climatic fluctuations.

Based on these objectives and the existing literature, we hypothesize that

**H1.**  *increasing mineral fertilization will lead to a predictable ecological succession, characterized by a decrease in oligotrophic indicator species and an increase in nitrophilous, competitive species, accompanied by a significant increase in biomass production.*

**H2.**  *in unfertilized control plots, interannual climatic variability—particularly variations in spring temperature and precipitation—will significantly alter floristic composition and the distribution of indicator species, independently of management effects.*

**H3.**  *in unfertilized control plots, biomass production will respond to interannual climatic variability and will be strongly correlated with specific climatic parameters during the growing season, reflecting functional ecosystem responses rather than compositional changes.*

These hypotheses were tested using complementary analytical approaches, including indicator species analysis, multivariate ordination techniques, and correlation analyses of biomass production and climatic variables, as detailed in the Materials and Methods and Results Sections.

By analyzing the distribution of indicator species in multivariate orderings, evaluating their relationship with climatic variables, and examining the influence of temperature and precipitation on production, this study provides a coherent perspective on the sensitivity of mountain grasslands to interannual changes. The results provide a solid basis for developing adaptive management strategies in the context of accelerated warming [53,54]. Overall, this study highlights both the vulnerability of these ecosystems and their capacity to respond in the short term, emphasizing the need for continuous monitoring of mountain grasslands.

## 2. Results

### 2.1. Effects of Mineral Fertilization Gradient on Indicator Species and Biomass Production

After 17 years of monitoring, mineral fertilization induced clear and consistent shifts in vegetation structure across the applied management gradient in the HNV grassland of the Apuseni Mountains. Increasing fertilization intensity promoted the replacement of oligotrophic plant communities by species assemblages dominated by competitive, nutrient-demanding grasses. These structural changes were accompanied by distinct indicator species patterns and were closely linked to increasing biomass production, reflecting the functional relationship between nutrient availability, species dominance, and ecosystem productivity.

#### 2.1.1. Indicator Species Response to Mineral Fertilization Levels

Indicator species analysis showed that the reference phytocoenosis (*Festuca rubra*–*Agrostis capillaris* type) had the highest number of indicator species. A total of 23 species with significant indicator values were identified for the reference variant (Table 1), most of which were classified as oligotrophic and oligomesotrophic species. Among these, 13 species exceeded a cover value of 0.5%. *Festuca rubra* showed the highest relative cover (19.5%), followed by *Anthoxanthum odoratum* (4.4%), *Plantago media* (3.8%), and *Trifolium repens* (3.3%). Three additional species slightly exceeded the 1% cover threshold: *Lotus corniculatus* (1.2%), *Thymus pulegioides* (1.2%), and *Scabiosa columbaria* (1.4%). *Briza media* and *Polygala vulgaris* occurred at very low cover values, close to the minimum inclusion threshold. Several additional species were present at similarly low abundances, including *Carex pallescens*, *Luzula multiflora*, *Gymnadenia conopsea*, and *Carlina acaulis*. In the variant where V2 (50N–25P_2_O_5_–25K_2_O kg ha^−1^) was applied, several species present in the control phytocoenosis were no longer identified as indicator species, including *Briza media*, *Carlina acaulis*, *Cerastium glomeratum*, *Gentiana lutea*, and *Thymus pulegioides*. The transition from the control variant to the first level of mineral fertilization (V2) was associated with the appearance of a new set of indicator species. The *Trisetum flavescens*–*Agrostis capillaris* (V2) grassland type included four indicator species, which are all characteristic of mesotrophic conditions generated by moderate mineral fertilization. Among these, *Trisetum flavescens* had the highest cover (23%), followed by *Alchemilla vulgaris* (8%), *Hypericum maculatum* (4%), and *Hieracium aurantiacum* (0.5%). Indicator values showed moderate to high species fidelity, with *Hypericum maculatum* displaying the strongest association with the respective fertilization level, followed by *Trisetum flavescens*, *Alchemilla vulgaris*, and *Hieracium aurantiacum*. For V2, these species reached maximum levels of participation in the vegetation cover, justifying their indicative value for this fertilization level. The disappearance of oligotrophic indicator species under moderate fertilization can be explained by increased nutrient availability, which enhances the competitive ability of faster-growing mesotrophic species and leads to increased canopy closure and light competition, thereby disadvantaging stress-tolerant species adapted to low-nutrient conditions.

An additional increase in the fertilization dose V3—100N–50P_2_O_5_–50K_2_O kg/ha led to the identification of a smaller number of indicator species for the applied fertilization gradient. The *Agrostis capillaris*–*Trisetum flavescens* (V3) grassland type is characterized by two species with indicative values associated with mesotrophic conditions specific to this level of fertilization. *Rumex acetosa* and *Veronica chamaedrys* occurred at similarly low cover values within the respective community (Table 1). The two species reached their maximum participation in V3 in relation to the other phytocoenoses, with similar indicator values: 37.1 for *Rumex acetosa* and 40.0 for *Veronica chamaedrys*. The reduced number of indicator species reflects a more simplified floristic structure than that of the previous variant.

The increase in fertilization dose to V4 (150N–75P_2_O_5_–75K_2_O kg ha^−1^) caused a further shift in floristic composition, expressed by the dominance of the *Agrostis capillaris* (V4) grassland type. This variant was characterized by two indicator species associated with mesotrophic-to-eutrophic conditions. *Agrostis capillaris* strongly dominated the grassland community under intensive fertilization, whereas *Taraxacum officinale* remained a subordinate component. Both species showed their strongest association with the highest fertilization level (V4), indicating a pronounced response to nutrient enrichment (Table 1). The pronounced dominance of *Agrostis capillaris* under high fertilization can be attributed to increased soil nitrogen availability, which favors fast-growing, competitive grasses with high nutrient uptake efficiency, leading to rapid cover closure and reduced light availability for subordinate species.

#### 2.1.2. Effects of Mineral Fertilization Gradients on Dry Matter Production in Mountain Grasslands

Dry matter yield increased consistently along the mineral fertilization gradient, showing a strong positive relationship between fertilization intensity and biomass production (Table 2). The lowest yields were recorded in the unfertilized control, while progressively higher fertilization levels resulted in marked increases in biomass production, with the maximum values observed under the highest fertilization intensity (V4). All fertilized variants produced significantly higher yields than the control, and yield differences between successive fertilization levels were statistically significant (*p* < 0.001; Table 3 and Table 4). Overall, the results demonstrate a clear productivity gradient driven by increasing mineral fertilization intensity, culminating in the highest biomass production under the V4 treatment.

#### 2.1.3. Indicator Species as Reflectors of the Fertilization Gradient and Yield Levels

**Figure 1:**
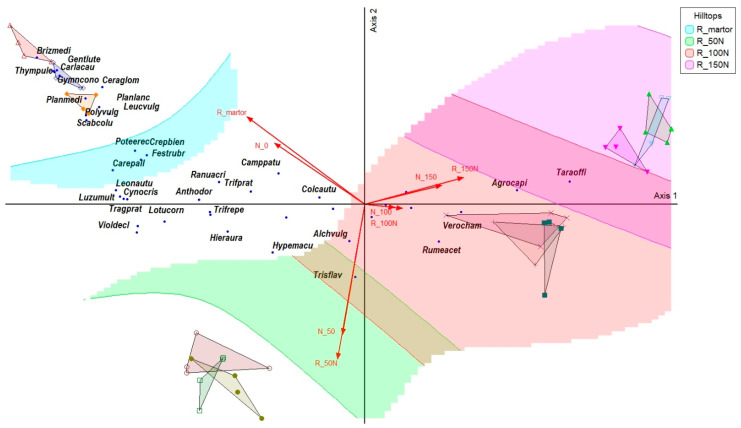
Principal coordinate analysis of grassland communities across nitrogen fertilization gradients. Legend: the colored polygons represent the four fertilization gradients applied to the grasslands: R_martor—control; R_50N—N_50_P_25_K_25_; R_100N—N_100_P_50_K_50_ R_150N—N_150_P_75_K_75_. The symbols indicate repetitions within each treatment, and the arrows represent nitrogen fertilization vectors, which describe the direction and intensity of changes in plant community composition: Brizmedi—*Briza media*; Thympule—*Thymus pulegioides*; Gentlute—*Gentiana lutea*; Carlcau—*Carlina acaulis*; Gymncono—*Gymnadenia conopsea*; Ceraglom—*Cerastium glomeratum*; Planmedi—*Plantago media*; Planlanc—*Plantago lanceolata*; Polyvulg—*Polygala vulgaris*; Scabcolu—*Scabiosa columbaria*; Poteerec—*Potentilla erecta*; Crepbien—*Crepis biennis*; Festrubr—*Festuca rubra*; Carepall—*Carex pallescens*; Leonautu—*Leontodon autumnalis*; Cynocris—*Cynosurus cristatus*; Luzumult—*Luzula multiflora*; Tragprat—*Tragopogon pratensis*; Lotucorn—*Lotus corniculatus*; Violdecl—*Viola declinata* Trifrepe—*Trifolium repens*; Trisflav—*Trisetum flavescens*; Alchvulg—Alchemilla vulgaris; Hypemacu—*Hypericum maculatum*; Hieraura—*Hieracium aurantiacum*; Rumeacet—*Rumex acetosa*; Verocham—*Veronica chamaedrys*; Colcautu—*Colchicum autumnale*; Camppatu—*Campanula patula*; Agrocapi—*Agrostis capillaris*; Taraoffi—*Taraxacum officinale*.

The PCoA ordination, overlaid with treatment polygons and harvest vectors, revealed a clear gradient in floristic composition associated with increasing fertilization intensity and grassland productivity. Along Axis 1, plant communities showed a gradual shift from the control variant (V1) toward higher fertilization levels (V2–V4). In the control variant (V1), oligotrophic species such as *Festuca rubra*, *Briza media*, *Anthoxanthum odoratum*, and *Cynosurus cristatus* were grouped on the left side of the ordination space, showing strong negative correlations with Axis 1 and being associated with low biomass production (2.79 t ha^−1^). With moderate fertilization (V2—N50P25K25), vegetation structure shifted toward the center of the ordination, where mesotrophic species including *Trisetum flavescens*, *Alchemilla vulgaris*, and *Hypericum maculatum* were positioned (Figure 1). These species showed intermediate correlations with the ordination axes and were associated with moderate biomass levels (3.67 t ha^−1^). Under higher fertilization intensity (V3—N100P50K50), plant communities moved further along the positive direction of Axis 1. Species such as *Rumex acetosa* and *Veronica chamaedrys* displayed positive correlations with Axis 1 and increased cover values, corresponding to higher productivity levels (4.80 t ha^−1^). The most pronounced shift occurred under the highest fertilization level (V4—N150P75K75), where the ordination was dominated by highly competitive species. *Agrostis capillaris* was positioned at the extreme positive end of Axis 1, showing a cover rate exceeding 60% and a strong positive correlation (r = 0.978) with Axis 1, and was associated with maximum biomass production (5.41 t ha^−1^). *Taraxacum officinale* showed a similar positioning, confirming its association with the highest fertilization intensity (Table 5).

Analyzed together, the ordination graph and the table of indicator species build a coherent ecological picture: as fertilization intensity increases, oligotrophic species are gradually replaced by mesotrophic and then nitrophilic species, and the structure of plant communities becomes increasingly simplified but more productive in terms of biomass production. The INDVAL, ADM, and r (Axis 1) values explain this gradual transition, and the association of each indicator species with the harvest level confirms the usefulness of floristic indicators for estimating the productive potential of HNV mountain grasslands.

Having established the profound impact of long-term mineral fertilization on floristic composition and biomass, the subsequent section delves into the second core objective of this study: to isolate and quantify the influence of interannual climatic variability on these grassland characteristics under conditions free from management-induced nutrient changes.

### 2.2. Climate Variability Effects on Indicator Species and Dry Matter Yield in Mountain Grasslands

Assessing the influence of climate change on mountain grasslands requires analyzing changes in plant communities under conditions free from trophic interventions so that the observed dynamics reflect climate variations alone. In the long-term experiment, this perspective can be documented by examining the control phytocoenoses (V1R1–V1R4), which represent the reference state of the natural ecosystem. The following section analyses the evolution of indicator species and dry matter production in the control variants in the experimental years 2015 (V1R1–V1R4), 2016 (V1R1–V1R4), and 2017 (V1R1–V1R4), in order to highlight the extent to which climatic fluctuations during that period influenced the structure and functioning of mountain grasslands. Floristic composition was assessed using the same sampling protocol, plot layout, cover estimation method, and observer in all experimental years, ensuring the full comparability of species composition data across years.

#### 2.2.1. Cluster Analysis of Species Responses to Climate Variability

**Figure 2:**
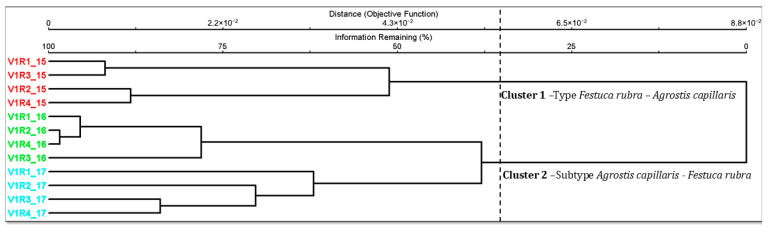
Floristic classification of the vegetation and changes in grassland type. Legend: V1—control; 15, 16, 17—the three experimental years—2015, 2016, 2017; R1–R4—replications.

The analysis of the floristic composition classification for the control variants in the experimental years 2015 (V1R1–V1R4), 2016 (V1R1–V1R4), and 2017 (V1R1–V1R4) showed clear differences between the variants and replicates, reflected in the formation of two distinct floristic groups. Cluster 1 includes all control variants from the 2015 experimental year, characterized by a floristic structure classified as *Festuca rubra*–*Agrostis capillaris* grassland. This cluster indicates a stable composition at the repetition level (V1R1–V1R4) and a clear differentiation from the next group. Cluster 2 includes the control variants from 2016 and 2017, associated with the *Agrostis capillaris*–*Festuca rubra* subtype. Within this cluster, the dendrogram highlights an internal subgroup, with the 2016 replicates in a compact group and the 2017 replicates occupying distinct positions but remaining within the same floristic cluster. The dendrogram indicates differences in floristic composition between the 2015 control variants and those of 2016–2017, reflected in them belonging to two different types of grasslands (Figure 2).

The Multi-Response Permutation Procedure (MRPP) analysis highlighted significant floristic differences between the three groups corresponding to the years 2015, 2016, and 2017. All comparisons showed *p*-values < 0.01 and positive A coefficients, indicating high internal homogeneity and clear separation between years. The highest values of internal agreement (A ≈ 0.44) were recorded for the T1–T2 and T1–T3 comparisons, suggesting pronounced floristic differences between 2015 and the other years of study. In ecological terms, the A values of this magnitude indicate a very strong effect size, reflecting highly distinct plant community compositions with minimal overlap between years. Overall, the results confirm that interannual variations in temperature and precipitation cause consistent and significant changes in the floristic composition of the control variant grasslands (Table 6). The strong internal homogeneity within each year’s cluster and clear separation between years indicate that annual climatic conditions significantly and distinctly shaped plant community structure, largely exceeding within-year plot variability.

#### 2.2.2. Climatic Gradient Indicator Species in Unfertilized Mountain Grasslands

The ISA applied to the control variants across the experimental years identified nine species with significant indicator values (*p* < 0.05), associated with distinct climatic conditions (Table 7). Species with year-specific indicator values were identified for each climatically differentiated study year.

The thermal and pluviometric conditions of 2015 were associated with the indicator species *Briza media*, *Festuca rubra*, *Alchemilla vulgaris*, and *Campanula patula*. The year 2016 was distinguished by the presence of *Pimpinella major* and *Thymus pulegioides*. In 2017, higher precipitation levels were associated with *Trisetum flavescens*, *Carex pallescens*, and *Centaurea pseudophrygia*, which were identified as indicator species for this climatic regime.

The ISA showed that the climatic variability in each experimental year clearly influenced the floristic composition of mountain grasslands, leading to the identification of specific indicator species for 2015, 2016, and 2017 based on the temperature and precipitation in that year. Individual analysis of indicator species is essential to understand how plants respond to climate change and to accurately characterize the adaptive capacity of plant communities in mountain ecosystems. Assessing the response of each species to the specific climatic conditions of each year provides valuable information on the sensitivity of the ecosystem and possible directions for changes in floristic composition. This approach is particularly important for developing realistic scenarios for the adaptation of mountain ecosystems to climate change, while also providing a basis for appropriate management measures capable of supporting the maintenance of biodiversity and functionality of grasslands in a changing climate. The PCoA confirmed a clear separation between the experimental years for the control variant, highlighting consistent changes in floristic composition associated with climate variability. Axis 1 explains 87.9% of the total variation and represents the main ecological gradient detected, corresponding to annual changes in the vegetation structure of grasslands (Table 8). Axis 2 contributed an additional 11.3% and further distinguished the floristic differences between years, with no overlap between groups, indicating a distinct response of plant communities to the climatic conditions specific to each experimental year.

The correlations between climatic variables, dry matter production, and ordination axes highlight the combined contribution of productivity and interannual climatic variability to the organization of ecological gradients captured by the PCoA. Axis 1 is primarily structured by the productivity gradient, with dry matter yield showing the strongest negative association (r = −0.952; r^2^ = 0.907). This indicates that variation in biomass production is a major driver of floristic separation along Axis 1, with negative values corresponding to communities with the highest biomass and positive values indicating less productive phytocoenoses (Table 9).

Temperature-related variables exhibited differentiated relationships with floristic structure. Temperatures in April and June (TM_Apr and TM_Jun) were negatively correlated with Axis 1 (r = −0.832 and r = −0.937), reflecting plant community sensitivity to temperature variability at the beginning of the growing season. In contrast, the May temperature (TM_May) showed a moderate positive correlation (r = 0.656), suggesting a distinct phenological influence on floristic variation along the upper part of Axis 1. Axis 2 was mainly associated with interannual climatic variability, being strongly correlated with mean annual temperature (r = −0.885; r^2^ = 0.783), indicating that differences between years are primarily reflected in overall thermal conditions rather than seasonal extremes.

Precipitation also contributed to the ordination structure. March and April precipitation (P_Mar and P_Apr) showed strong negative correlations with Axis 2 (r = −0.886 for both), while precipitation in May and June (P_May and P_Jun) was negatively correlated with Axis 1 (r = −0.615 and r = −0.491), emphasizing the role of spring rainfall in structuring less productive plant communities. It should be noted that several climatic variables are partially intercorrelated; therefore, the observed relationships reflect combined climatic influences and overall ecosystem sensitivity to year-to-year climatic variability rather than independent effects of individual parameters. Given the relatively short temporal window of climatic observations (2015–2017), these results should be interpreted as responses to interannual variability within the study period.

**Figure 3:**
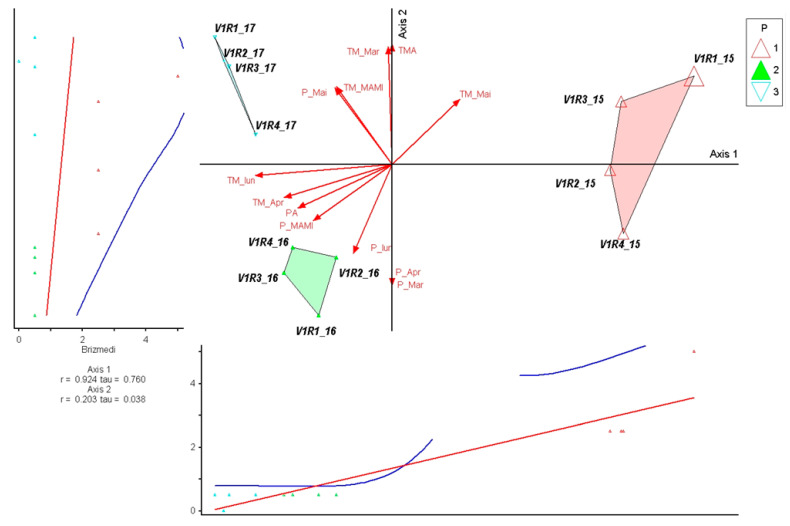
PCoA ordination of control plots and *Briza media* response in relation to climatic variation (2015–2017). Triangles represent individual plots, colored by year (1—2015, 2—2016, 3—2017). Red arrows indicate climatic variables fitted onto the ordination space, with arrow length reflecting the strength of correlations. Blue and red lines represent fitted trend lines describing the response of *Briza media* along the main ordination axis.

The indicator species *Briza media* showed a clear association with Axis 1 (r = 0.924; τ = 0.760), reflecting its sensitivity to the climatic gradient that differentiated 2015 from 2016 to 2017. The species is favored by conditions with high water supply and moderate temperatures at the beginning of the season, which explains its higher values in 2015. A very weak correlation with Axis 2 (r = 0.203) indicates that late rainfall variations contribute marginally to its distribution. The low levels in 2016 corresponded to a water deficit and higher spring temperatures, and in 2017, only partial recovery was observed, supported by rainfall in May. This pattern confirms the role of *B. media* as an indicator species for the climatic conditions characteristic of 2015 (Figure 3).

**Figure 4:**
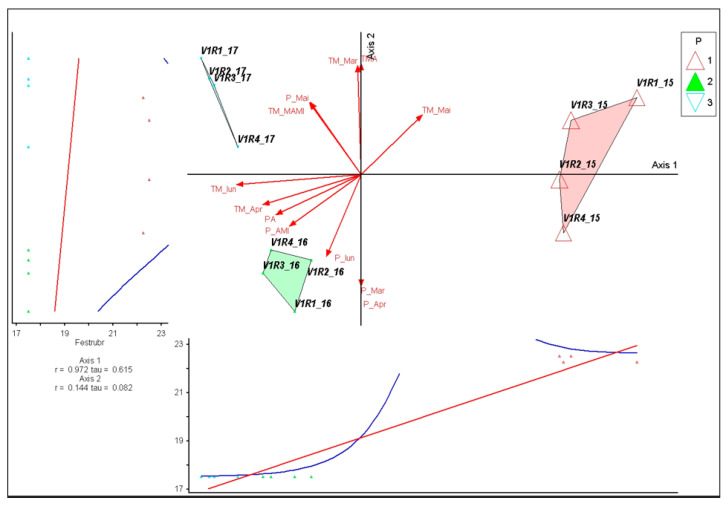
PCoA ordination of control plots and *Festuca rubra* response in relation to climatic variation (2015–2017). P—plots; 1—year 2015, 2—year 2016, 3—year 2017. Red arrows represent climatic variables; blue and red lines indicate fitted trend lines; different colored triangles represent plots and years.

*Festuca rubra* showed a strong association with Axis 1 (r = 0.971; τ = 0.574). The high correlation values show that the species is characteristic of colder climatic intervals with low productivity, where the spring temperature vectors (TM_Mar, TM_Apr) have low intensities. The very weak correlation with Axis 2 (r = 0.155) confirms that precipitation variations are not a major determinant of its abundance. The distribution associated with 2015 shows that *F. rubra* remains relatively stable in temperate and less productive conditions, justifying its status as an indicator species for this year in the ISA. This pattern suggests moderate adaptability to climatic fluctuations, with the species maintaining its constancy in colder conditions but showing noticeable sensitivity when spring temperatures rise (Figure 4).

**Figure 5:**
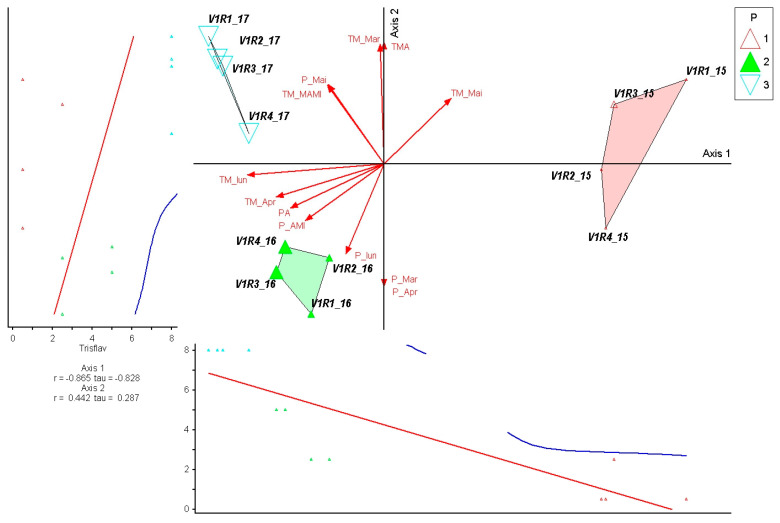
PCoA ordination of control plots and *Trisetum flavescens* response in relation to climatic variation (2015–2017). P—plots; 1—year 2015, 2—year 2016, 3—year 2017. Red arrows represent climatic variables; blue and red lines indicate fitted trend lines; different colored triangles represent plots and years.

The species *Trisetum flavescens* shows a strong correlation with Axis 1 (r = −0.865; τ = −0.828), indicating that its abundance variation is mainly determined by the climatic gradient represented by monthly average temperatures. This suggests *Trisetum flavescens* act as a key indicator for early-growing-season temperature shifts in these mountain grasslands, potentially reflecting its sensitivity to thermal conditions. The vectors associated with temperatures in March, April, and May consistently align with the direction of the axis, suggesting a high sensitivity of the species to early warming in the growing season. The moderate correlation with Axis 2 (r = 0.442; τ = 0.287) reflects a complementary response to precipitation dynamics, but with a secondary influence on the structure of the variation (Figure 5).

**Figure 6:**
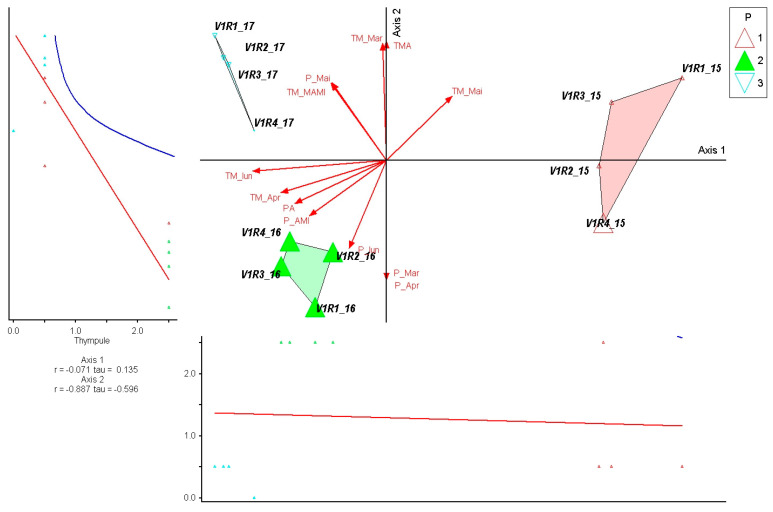
PCoA ordination of control plots and *Thymus pulegioides* response in relation to climatic variation (2015–2017). P—plots; 1—year 2015, 2—year 2016, 3—year 2017. Red arrows represent climatic variables; blue and red lines indicate fitted trend lines; different colored triangles represent plots and years.

The species *Thymus pulegioides* shows a very strong correlation with Axis 2 (r = −0.887; τ = −0.596), indicating that its abundance variation is predominantly influenced by precipitation dynamics, especially by the rainfall regime in April–June. The negative orientation of the species on this axis shows a preference for drier conditions, correlated with the vectors describing precipitation reductions, which explains the close association with 2016, characterized by a pronounced water deficit. The very weak correlation with Axis 1 (r = −0.071; τ = 0.135) shows that temperature plays a secondary role in determining its distribution (Figure 6).

**Figure 7:**
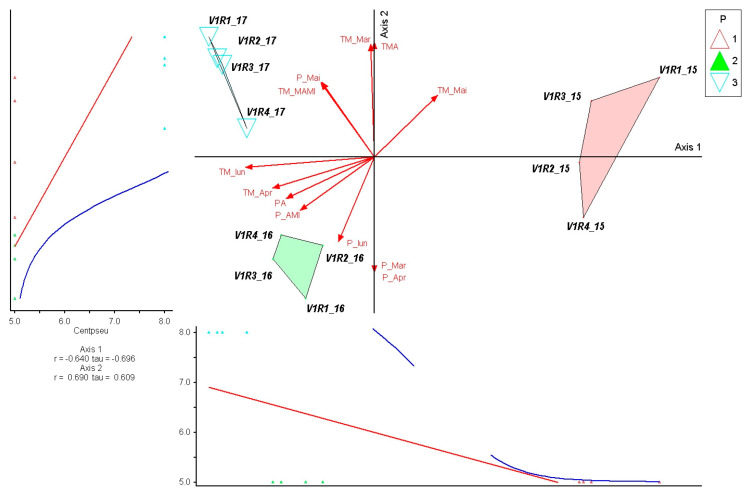
PCoA ordination of control plots and *Centaurea pseudophrygia* response in relation to climatic variation (2015–2017). P—plots; 1—year 2015, 2—year 2016, 3—year 2017. Red arrows represent climatic variables; blue and red lines indicate fitted trend lines; different colored triangles represent plots and years.

The *Centaurea pseudophrygia* species shows a strong positive correlation with Axis 2 (r = 0.679; τ = 0.609) and a significant negative correlation with Axis 1 (r = −0.648; τ = −0.696). In the ordination, its position clearly correlates with the rainfall vectors (P_Mar, P_Apr, P_Jun), indicating a strong association with spring–early summer water supply. The negative correlation with Axis 1 shows that temperature variations have a secondary influence on its distribution. According to the IndVal analysis (Maxgrp = 3), the species is mainly associated with the year 2017, characterized by higher precipitation (Figure 7).

**Figure 8:**
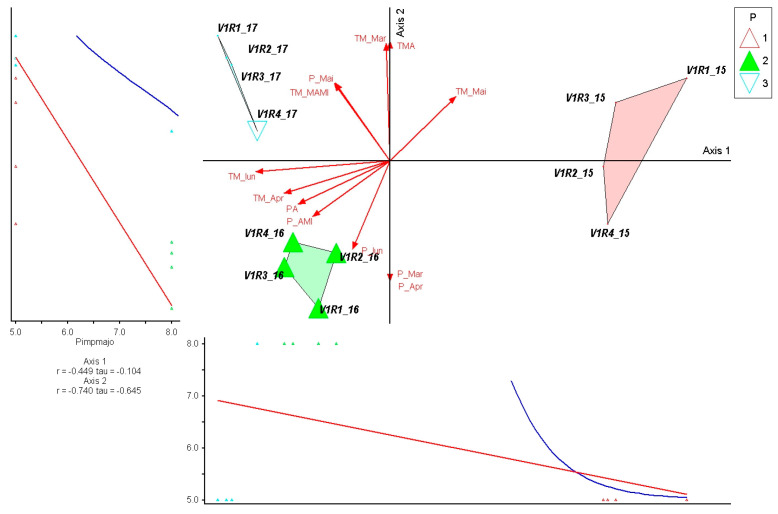
PCoA ordination of control plots and *Pimpinella major* response in relation to climatic variation (2015–2017). P—plots; 1—year 2015, 2—year 2016, 3—year 2017. Red arrows represent climatic variables; blue and red lines indicate fitted trend lines; different colored triangles represent plots and years.

The species *Pimpinella major* shows a strong negative correlation with Axis 2 (r = −0.740; τ = −0.645), indicating high sensitivity to precipitation dynamics in spring–early summer. Its position in the ordination shows a clear association with the drier conditions of 2016, in line with the direction of the rainfall vectors. In contrast, the low correlation with Axis 1 (r = −0.449; τ = −0.104) confirms that temperature variations have a secondary influence on its distribution. In the graph, the species is exclusively concentrated in the 2016 group, where the water deficit favored its presence, while in 2015 and 2017, the values are low, reflecting a limited tolerance to high-rainfall regimes. Overall, *P. major* emerges as an indicator of drier years, with a predictable ecological response to water availability at the beginning of the growing season (Figure 8).

**Figure 9:**
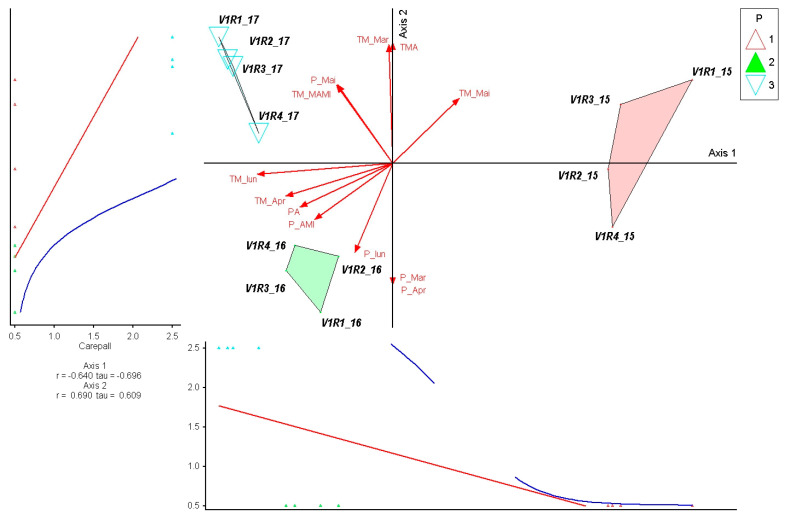
PCoA ordination of control plots and *Carex pallescens* response in relation to climatic variation (2015–2017). P—plots; 1—year 2015, 2—year 2016, 3—year 2017. Red arrows represent climatic variables; blue and red lines indicate fitted trend lines; different colored triangles represent plots and years.

The species *Carex pallescens* showed a strong negative correlation with Axis 1 (r = −0.640; τ = −0.696), indicating an ecological response that is mainly determined by the rainfall gradient from March to June. The species’ position towards the negative end of this axis shows a preference for drier conditions, which explains the highest values recorded in 2017—a year characterized by rainfall deficit in the first part of the season. The positive correlation with Axis 2 (r = 0.690; τ = 0.609) suggests a secondary influence of early temperatures, but their effect is less pronounced compared to precipitation. In the ordination, *C. pallescens* is concentrated in the 2017 group, with intermediate values in 2016 and low values in 2015, reflecting a low tolerance to high-rainfall regimes and an adaptation to drier years. Thus, the species emerges as an indicator of low-humidity conditions at the beginning of the growing season (Figure 9).

**Figure 10:**
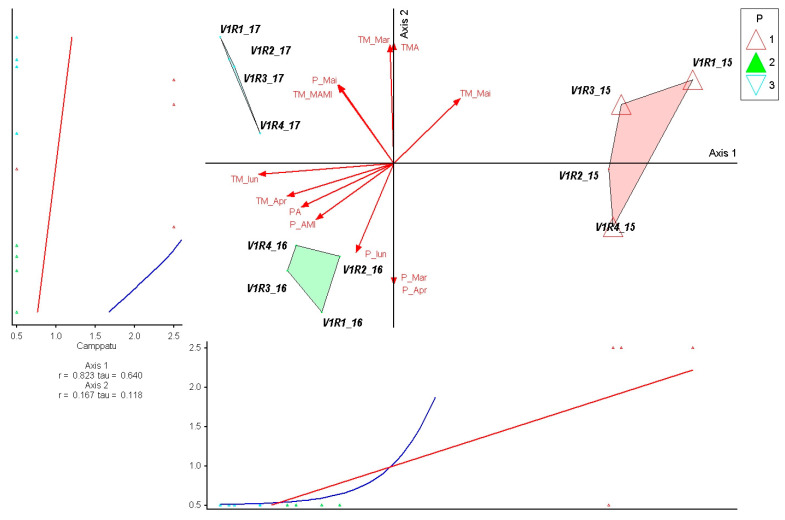
PCoA ordination of control plots and *Campanula patula* response in relation to climatic variation (2015–2017). P—plots; 1—year 2015, 2—year 2016, 3—year 2017. Red arrows represent climatic variables; blue and red lines indicate fitted trend lines; different colored triangles represent plots and years.

The species *Campanula patula* shows a very strong and positive correlation with Axis 1 (r = 0.823; τ = 0.640), indicating a strong ecological response determined by the rainfall gradient in March–June, which is represented on this axis. The species’ position in the positive area of Axis 1 reflects its preference for wetter conditions, as confirmed by the concentration of high values in 2015, when rainfall in the first part of the growing season was higher than in other years. The weak correlation with Axis 2 (r = 0.167; τ = 0.118) indicates that early temperatures have a reduced effect on the distribution of the species. In the ordination, *C. patula* is grouped compactly in 2015, while in 2016, the values are moderate and dispersed, and in 2017, the species is present only with low values, suggesting a high sensitivity to dry conditions. Overall, the species emerges as an indicator of wetter years, responding favorably to precipitation at the beginning of the growing season (Figure 10).

**Figure 11:**
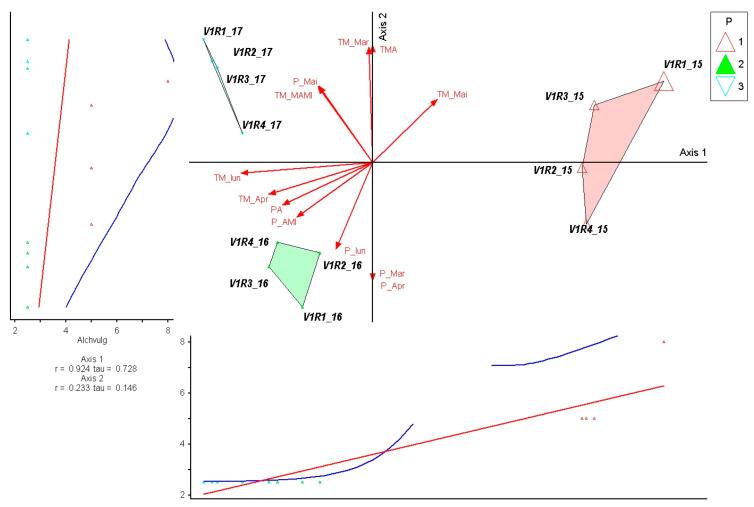
PCoA ordination of control plots and *Alchemilla vulgaris* response in relation to climatic variation (2015–2017). P—plots; 1—year 2015, 2—year 2016, 3—year 2017. Red arrows represent climatic variables; blue and red lines indicate fitted trend lines; different colored triangles represent plots and years.

*Alchemilla vulgaris* shows a very strong and positive correlation with Axis 1 (r = 0.924; τ = 0.728), highlighting a major dependence on the temperature gradient in March–May. The high values in 2015 confirm the species’ preference for warmer conditions at the beginning of the growing season. The low correlation with Axis 2 (r = 0.233; τ = 0.146) shows a secondary effect of early thermal variations on distribution. In the ordination, the species is concentrated in the 2015 group, while in 2016, abundance decreases considerably, suggesting a reduced tolerance to wetter regimes. In 2017, moderate values reflect an intermediate response to accentuated thermal conditions. Overall, *A. vulgaris* emerges as an indicator of warmer years, which are sensitive to excess precipitation in the first part of the season (Figure 11).

#### 2.2.3. Climatic Effects on Biomass Yield Dynamics to the Control Variant

The PCoA analysis overlaid with climatic vectors and biomass yield highlights how thermal and pluviometric fluctuations during 2015–2017 influenced dry matter production in the mountain grassland ecosystem. The yield vector is strongly oriented towards the negative direction of Axis 1, indicating a very strong correlation (r = −0.952; r^2^ = 0.907). This demonstrates that Axis 1 represents the main ecological gradient explaining variation in biomass production.

**Figure 12:**
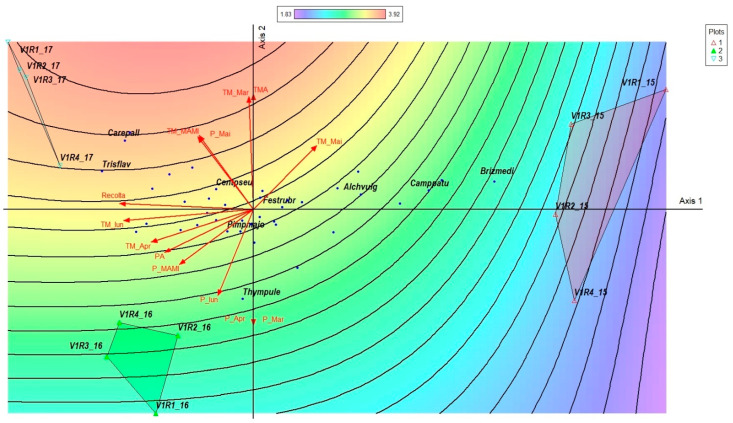
Dry matter yield response to interannual climatic variability in the unfertilized control grassland (V1) principal coordinate analysis (PCoA) illustrating the relationship between dry matter yield and interannual climatic variability (temperature and precipitation, March–June) for the unfertilized control variant (V1) during the study period (2015–2017). Red vectors indicate the direction and strength of correlations with monthly temperature (TM) and precipitation (P) variables. The color gradient and contour lines represent the fitted surface of dry matter yield. Plot groupings correspond to individual study years. P—plots; 1—year 2015, 2—year 2016, 3—year 2017.

The unfertilized control replicates were separated along Axis 1 according to interannual climatic conditions and associated yield levels. The 2015 replicates clustered on the positive end of Axis 1 in the low-yield region, aligning with vectors indicating cooler early-season temperatures (TM_Mar, TM_Apr) and reduced precipitation. In contrast, the 2016 replicates formed a compact group on the negative side of Axis 1, associated mainly with higher early-spring precipitation (P_Mar, P_Apr, P_MAMI) and intermediate yields. The 2017 replicates occupied the upper-left ordination space and aligned with higher early-season temperatures (TMA, TM_Mar, TM_Apr), together with June precipitation (P_Iun), corresponding to the highest yield levels. Overall, these patterns indicate that yield variability across years was primarily structured by the climatic gradient captured by Axis 1, integrating early-season temperature and spring–early summer precipitation effects. Interannual variation in dry matter yield of the unfertilized control plots was closely associated with climatic conditions, as illustrated by the PCoA ordination (Figure 12).

## 3. Discussion

### 3.1. Ecological Responses to Fertilization and Floristic Succession

Long-term mineral fertilization induced a predictable ecological trajectory in the HNV mountain grassland by altering competitive hierarchies through sustained increases in nutrient availability. Enhanced nitrogen supply favored species with acquisitive growth strategies, characterized by rapid biomass accumulation and high nutrient uptake efficiency, while oligotrophic specialists typical of *Festuca rubra*–*Agrostis capillaris* grasslands declined due to reduced competitive ability [15,22,23]. As fertilization intensity increased, mesotrophic and subsequently nitrophilous species expanded as a result of intensified light interception by taller grasses, increased shading at the ground layer, and more effective monopolization of soil nitrogen by fast-growing species [55,56,57,58]. These processes reflect the classical fertilization–diversity trade-off observed in European mountain grasslands, where productivity gains are accompanied by competitive exclusion, floristic homogenization, and a decline in species adapted to low-nutrient conditions [59,60,61].

The high number of indicator species recorded in the unfertilized control confirms the high ecological integrity of low-input HNV systems, consistent with findings from Apuseni grasslands managed traditionally with minimal external inputs [62,63]. The high number of indicator species recorded in the reference phytocoenosis reflects the ecological stability of unfertilized grasslands and the persistence of oligotrophic and oligomesotrophic species. Such species assemblages are typically associated with low nutrient input and long-term extensive management, underlining the conservation value of high-nature-value mountain grasslands [24,38]. Under moderate fertilization (V2–V3), the emergence of *Trisetum flavescens*, *Alchemilla vulgaris*, or *Hypericum maculatum* reflects a shift toward mesotrophic assemblages, similar to transitional communities observed in Romanian and Central European mountain meadows exposed to low–medium nutrient inputs [64,65,66,67,68,69,70].

At the highest fertilization level (V4), the dominance of *Agrostis capillaris* and the decline of oligotrophic species reflect fundamental shifts in competitive interactions within the plant community. Increased nitrogen availability favors fast-growing grasses with high nutrient uptake efficiency and dense cover formation, enhancing light interception and reducing light availability at the soil surface [15,59]. This process limits the establishment and persistence of low-stature, slow-growing oligotrophic species that are adapted to nutrient-poor conditions. In addition, intensified belowground competition for nitrogen further disadvantages species with conservative resource-use strategies. Together, these mechanisms drive floristic homogenization and reduced structural complexity, a pattern widely reported in long-term fertilization experiments where productivity gains are achieved at the expense of biodiversity and ecosystem stability [71,72,73,74,75].

Overall, the fertilization gradient confirms that nutrient enrichment remains one of the strongest drivers of floristic reorganization in HNV mountain grasslands. The convergence of our results with research from Apuseni and across Europe reinforces the sensitivity of oligotrophic communities to even moderate nutrient inputs and highlights the diagnostic value of indicator species for detecting nutrient-induced shifts in grassland functioning [24,43,76].

### 3.2. Climatic Variability as a Driver of Short-Term Community Reorganization

In the unfertilized control plots, where management influences are absent, interannual climatic variability emerged as the dominant driver of floristic dynamics. The separation of the three experimental years in ordination space indicates rapid community reorganization in response to variations in spring temperature and precipitation [37,51,52]. Similar patterns have been reported in mountain grasslands of the Alps and Carpathians, where cooler and drier springs were associated with reduced grass growth and increased persistence of stress-tolerant species, while warmer and wetter years promoted shifts toward more productive, mesotrophic assemblages [77,78,79,80]. These responses are characteristic of mountain ecosystems, where shallow soils, short growing seasons, and steep climatic gradients amplify the effects of annual weather variability. Indicator species identified for each year reflect clear ecological mechanisms. Species associated with cooler, moisture-rich conditions (*Briza media*, *Festuca rubra*, *Campanula patula*) dominated in 2015, consistent with their known affinity for mesic, oligotrophic environments [22,41,81,82,83,84,85,86]. In contrast, warmer and drier 2016 conditions favored drought-tolerant species such as *Thymus pulegioides* and *Pimpinella major*, which commonly increase during years characterized by reduced early-season rainfall [87,88,89]. In 2017, increased rainfall and warmer early-summer temperatures supported species such as *Trisetum flavescens* and *Centaurea pseudophrygia*, matching patterns observed in mountain ecosystems where warmer, wetter periods promote mesotrophic species expansion [90,91]. Altogether, these species-specific patterns illustrate the nuanced ecological strategies through which grassland plants cope with changing climates, strategies that determine community-level resilience or sensitivity to future climatic shifts [92,93,94]. These short-term shifts reinforce the idea that even floristically stable grasslands can reorganize within 1–2 years when climatic drivers change—an ecological behavior documented in various Carpathian and alpine systems [63,95,96]. Biomass followed the same climatic gradients, with low productivity in cold/dry years and peak yields during warm, well-watered seasons. This strong climate–productivity link confirms observations from long-term monitoring in Apuseni grasslands, where early-season temperatures and spring rainfall consistently shape annual forage output [40,97,98,99]. Together, these results highlight the dual pressures acting upon mountain grasslands: nutrient-driven long-term succession and climate-driven short-term fluctuations. Both processes reduce ecological predictability and may accelerate transitions toward more homogenized, less resilient systems in the face of ongoing climate change [100,101].

### 3.3. Practical Implications for the Management and Conservation of Mountain HNV Grasslands

The combined evidence from fertilization and climate-driven dynamics provides valuable insights for designing adaptive management strategies in HNV landscapes of the Apuseni Mountains. First, the strong responsiveness of oligotrophic species to nutrient enrichment highlights the need to maintain low-input systems—confirming recommendations from regional studies emphasizing that minimal fertilization preserves biodiversity, structural complexity, and habitat value [23,24,25,102]. Second, climate-sensitive indicator species identified in this study can be incorporated into local monitoring schemes. Species such as *Briza media* (wet–cold years) or *Thymus pulegioides* (dry–warm years) act as early-warning signals of climatic deviations, complementing meteorological data and helping managers anticipate changes in productivity, forage quality, and habitat suitability. Similar bioindicator-based approaches were recommended for Apuseni grasslands in works addressing landscape dynamics and traditional management systems [69,88,98]. Third, the observed sensitivity of biomass to climatic variability underscores the need for flexible mowing schedules. In years with delayed growth or early-season drought, fixed mowing dates may reduce both yield and forage quality, while adaptive timing—aligned with phenological development—could optimize production without compromising biodiversity [103,104].

Finally, the results support the conservation relevance of maintaining mosaic management at landscape scale. The diversity of microhabitats generated through heterogeneous use (low-input hay meadows, lightly grazed areas, occasional mulching) has been shown to enhance landscape resilience and species persistence in HNV systems [105,106].

### 3.4. Limitations and Future Research Perspectives

This study is based on three years of climatic variability, which, although sufficient to detect strong ecological responses, does not capture the full amplitude of long-term climate trends. Extending monitoring over a longer period would allow the identification of nonlinear or delayed responses. Additionally, the experiment is restricted to a single altitudinal belt; future studies could compare multiple elevations to quantify the interaction between altitude, microclimate, and management. While indicator species analysis and PCoA capture community-level patterns effectively, integrating functional traits (CSR strategies, rooting depth, phenology) would improve mechanistic understanding of species responses to warming and fluctuating precipitation. Incorporating remote sensing (drone or satellite data) could further enhance the capacity to upscale species-level signals to landscape-scale patterns—a research direction already emerging in Apuseni grassland studies [107]. Overall, future work should combine traditional floristic monitoring with functional, physiological, and remote-sensing approaches to better predict how HNV grasslands will respond to accelerating climate change and to design evidence-based conservation strategies.

## 4. Materials and Methods

### 4.1. Study Area

The experiment was conducted in Gârda de Sus, Alba County, Apuseni Mountains (Western Carpathians, Romania; 46°29′26.4″ N 22°48′53.7″ E), on a semi-natural permanent grassland located at an average altitude of 1130 m with a slope of approximately 5%. The grassland is situated within the Natura 2000 network, inside the ROSCI0002 Apuseni site, and corresponds to habitat type 6520—mountain hay meadows. This habitat type is known for its exceptional floristic richness and long-term cultural landscape continuity, as documented for Apuseni grasslands [70,88,104]. The site hosts a diverse assemblage of characteristic mountain species, including several of conservation interest such as *Lilium jankae*, *Allium victoriale*, *Centaurea kotschyana*, *Trollius europaeus* and *Anemone narcissifolia*, which underline the ecological sensitivity of the system to nutrient inputs and management practices. The soil had the typology of red, weakly skeletal preluvosol, with predominantly southeast exposure, characterized by moderate fertility and a low content of humus and nutrients, which determined its limited natural productivity. At the beginning of the long-term experiment, the soil was characterized by low nutrient availability. The surface horizon showed a high organic matter content (15.42%, including non-humified organic material) and a total nitrogen content of 0.75%, both decreasing sharply with depth. Soil reaction was moderately acidic in the upper horizons (pH 5.21–5.54). Available phosphorus and potassium contents were low, with P ranging from 2 to 10 ppm and K from 23 to 109 ppm, indicating oligotrophic condition [108]. The multiannual average temperature was 5.1 °C [59], and the annual precipitation amounted to approx. 1042 mm. Management consisted of annual mowing, carried out at the beginning of July, and the resulting grass was removed from the experimental plots. The analyzed data covered three experimental years (2015–2017), but reflected the cumulative effect of mineral fertilization applied annually since 2001, the year of the initiation of the experiment [72,109,110,111].

Regarding the temperature situation recorded at the Ghețari station, in the last 17 years, the following aspects can be observed: the multiannual average was around 5.80 °C, with a maximum value of 7.7 °C recorded in 2012 [23] and 2015 (Table 10), respectively, and a minimum value of 3.2 °C recorded in 2005 [23]. Table 10 also shows the increasing trend of the average annual temperature, especially in the period 2015–2017 (experimental years). Therefore, the context of climatological changes in the study area brings important effects/changes on the floristic diversity of the oligotrophic meadows in the Apuseni Mountains. Climatic data were recorded by an automatic meteorological station located directly within the experimental field, at the same elevation as the study plots, ensuring high data accuracy and eliminating spatial or altitudinal discrepancies.

Regarding the average annual precipitation values recorded at the Ghețari weather station, it was found that the multiannual average (over 17 years) had the value of 1042.1 mm, with the maximum being recorded in 2001 (1553 mm) and the minimum recorded in 2012 (687 mm), which was considered the driest year in our study area. Comparing the 3 experimental years (2015–2017 period) with the multiannual average, it can easily be seen that the decreasing trend in precipitation values is also complemented by the increasing temperature values (Table 11); therefore, a continuously changing picture is foreseen from a climatological point of view.

### 4.2. Experimental Design

A long-term experiment established in 2001 was used to analyze vegetation trends. A completely randomized block design was applied, comprising four treatment variants with four replications, resulting in a total of 16 permanent adjacent plots, each covering an area of 10 m^2^ (2 × 5 m). The plots were arranged on a relatively uniform grassland surface along the natural slope to minimize topographic variability. Within each block, experimental plots were adjacent and no buffer strips were established between treatment variants; however, a buffer zone of 1 m was maintained between blocks (replicates) to reduce potential edge effects and nutrient transfer. Fertilizer application was carefully controlled to avoid cross-contamination between adjacent plots. The treatments were as follows (kg ha^−1^): V1—control (unfertilized), V2—N50P25K25 (N 50 kg ha^−1^, P_2_O_5_ 25 kg ha^−1^, K_2_O 25 kg ha^−1^), V3—N100P50K50 (N 100 kg ha^−1^, P_2_O_5_ 50 kg ha^−1^, K_2_O 50 kg ha^−1^), and V4—N150P75K75 (N 150 kg ha^−1^, P_2_O_5_ 75 kg ha^−1^, K_2_O 75 kg ha^−1^).

Mineral fertilizers were administered annually, in early spring, before the start of active vegetation. Granular NPK 15:15:15 was used, while nitrogen supplementation was provided as granular ammonium nitrate, supplying nitrogen in both nitrate (NO_3^−^_) and ammonium (NH_4^+^_) forms, with nitrate representing the readily soluble fraction. They were applied once a year in each experimental variant.

Aboveground biomass was harvested once per year by mowing the entire surface of each experimental plot (10 m^2^), with four replicates per treatment. Mowing was performed using a motorized mower, ensuring a uniform cutting height of approximately 4 cm. No subsampling quadrats were used, as biomass was collected from the full plot area.

### 4.3. Floristic Composition

**Figure 13:**
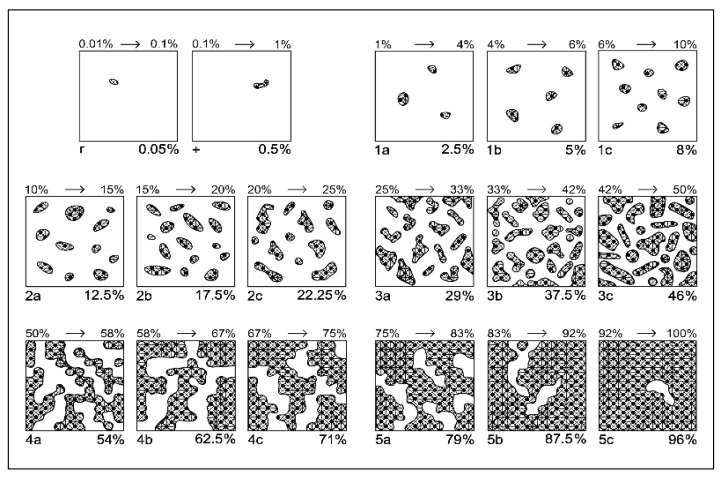
Modified Braun–Blanquét scale for grasslands based on species coverage (after [112]). Legend: 1 to 5 indicate the class of coverage; a, b, c indicate the sub-note of each class.

Floristic data were recorded annually during the 2015–2017 period, representing the vegetation response after more than 15 consecutive years of differentiated mineral fertilization (initiated in 2001). In each experimental year, species composition was assessed in all plots using the Braun–Blanquet method (Table 12, Figure 13), applying the extended cover–abundance scale adapted for mountain grasslands according to the methodological refinements introduced by [112]. Vegetation cover was assessed using the Braun–Blanquet cover–abundance method, based on modified classes that were converted into percentage cover values for each plant species.

This approach ensured consistent, high-resolution documentation of both dominant and low-abundance species across years, allowing the evaluation of cumulative fertilization effects together with short-term climatic variability. Mowing was carried out at the optimal time (when the grasses were in the phenological flowering phase), and the vegetal material was removed outside the experiment. Prior to the establishment of the long-term experiment in 2001, the grassland was managed under a traditional extensive regime. Management consisted of a single annual mowing followed by low-intensity autumn grazing (0.2–0.4 LU ha^−1^), without any application of mineral fertilizers; nutrient inputs were supplied solely through the presence of grazing livestock, a typical practice in the Apuseni Mountains [89,113,114].

### 4.4. Data Analysis

Multivariate analyses were performed using PC-ORD v.7 [99,100], a statistical package widely used in community ecology for classification, ordination, and testing differences among groups, and recognized as highly effective for examining complex floristic structures. To classify floristic types, cluster analysis was applied using the Sørensen (Bray–Curtis) index and the UPGMA agglomeration method, which are both considered standard approaches in plant community analysis [49]. The dendrogram cut level was set to retain approximately 80% of the information, enabling the identification of groups with clear ecological and phytosociological relevance along the mineral fertilization gradient and in relation to the climatic variability observed during the study years. To highlight the trophic gradient and differentiate grassland types according to treatment, principal coordinate analysis (PCoA) was used, based on Bray–Curtis distances—an established method in plant community ecology for comparing floristic and microbial compositions. The use of PCoA allowed a reproducible interpretation of patterns generated both by long-term fertilization inputs and by interannual climatic variation. This ordination approach is well supported in recent studies assessing fertilization-induced changes as well as environmentally driven dynamics in plant, fungal, and bacterial communities. To identify the species characteristic of each fertilization regime and to evaluate their sensitivity to annual climatic differences, indicator species analysis (ISA) was applied according to the methodology of [22,38,45]. Indicator species analysis (ISA) was used to identify species characteristic of each fertilization regime, following the method described by Dufrêne and Legendre [115], as implemented in PC-ORD version 7. Differences in species composition among fertilization treatments and among years were further tested using Multi-Response Permutation Procedures (MRPPs), based on Bray–Curtis distance matrices, with statistical significance assessed using 999 permutations. This non-parametric approach was applied to evaluate the strength and significance of group separation in floristic composition. The method combines species fidelity and relative abundance within each treatment to calculate an indicator value (IV, %), ranging from 0 to 100, with higher values indicating stronger association with a given treatment. The statistical significance of indicator values was tested using a Monte Carlo permutation procedure with 999 random permutations. Species with significant indicator values (*p* < 0.05) were considered reliable indicators of specific fertilization regimes. This approach is widely applied to detect vegetation–environment relationships and structural changes in plant communities [115]. This procedure combines the fidelity and constancy of species occurrence within treatments, generating an indicator importance value (IV, %) for each species. The statistical significance of IV values was assessed using permutation tests (*n* = 999), a method widely recognized for its accuracy in highlighting flora–environment relationships and detecting structural changes triggered by fertilization and climatic variability. The analysis of biomass production was performed using analysis of variance (ANOVA) to evaluate significant differences among the experimental treatments [116,117,118,119,120]. Prior to applying the ANOVA test, the data were checked to ensure that the assumptions of normality and homogeneity of variances [42,75,120,121,122,123,124,125].

## 5. Conclusions

This long-term experiment demonstrates that high-nature-value mountain grasslands are highly sensitive to both mineral fertilization and interannual climatic variability. Increasing nutrient inputs consistently promoted a shift from species-rich oligotrophic communities toward simplified, mesotrophic-to-eutrophic grassland types, accompanied by a loss of indicator species characteristic of low-input systems. At the same time, unfertilized grasslands responded rapidly to short-term climatic fluctuations, confirming that climate variability alone can significantly restructure floristic composition and productivity in mountain environments.

These findings highlight important implications for European grassland management and conservation policies. Maintaining low-input management regimes is essential to preserve biodiversity and ecological resilience in HNV grasslands, particularly under increasing climatic uncertainty. Agri-environmental schemes under the Common Agricultural Policy should prioritize fertilization limits, climate-adaptive management, and species-based indicators to safeguard ecosystem functions. The indicator species framework developed in this study provides a practical tool for monitoring grassland condition and supporting evidence-based management of mountain grasslands in the Carpathian region and other European highland systems. Future research should extend similar long-term experimental approaches across multiple mountain regions to assess the generality of these responses and to better understand how interannual climatic variability interacts with management practices over longer temporal scales.

## Figures and Tables

**Table 1:** Indicator species for the applied mineral fertilization treatments (2015–2017).

Species	Variant (V)	INDVAL	Mean	Std. Dev.	Semnif. Signif	N	ADM (%)
*Anthoxanthum odoratum* L. s. str.	V1	48.6	29.9	3.7	***	X	4.4
*Briza media* L.	V1	83.3	15.3	6.3	***	3	0.7
*Cynosurus cristatus* L.	V1	46.3	18.4	5.3	***	4	0.5
*Festuca rubra* L.	V1	66.2	33.0	3.8	***	X	19.5
*Carex pallescens* L.	V1	52.2	20.9	5.0	***	4	0.5
*Luzula multiflora* (Ehrh.) Lej	V1	50.0	21.3	4.9	***	3	0.5
*Lotus corniculatus* L	V1	54.9	24.6	5.9	***	4	1.2
*Trifolium pratense* L.	V1	47.7	32.1	4.1	***	6	2.6
*Trifolium repens* L.	V1	47.1	31.6	3.7	***	6	3.3
*Carlina acaulis* L.	V1	58.3	11.8	5.3	***	2	0.5
*Cerastium glomeratum* Thuill	V1	51.0	12.5	5.3	***	4	0.5
*Gentiana lutea* L.	V1	58.3	11.6	5.4	***	2	0.5
*Gymnadenia conopsea* (L.) R. Br. s. l.	V1	83.3	13.6	5.4	***	3	0.5
*Leontodon autumnalis* L.	V1	45.0	16.8	5.4	***	5	0.5
*Leucanthemum vulgare* Lam. s. str.	V1	75.4	21.4	5.7	***	3	2.3
*Plantago lanceolata* L.	V1	87.5	21.7	6.2	***	X	2.6
*Plantago media* L.	V1	90.2	22.5	6.3	***	3	3.8
*Polygala vulgaris* L.	V1	80.9	17.0	6.3	***	2	0.7
*Potentilla erecta* (L.) Raeusch.	V1	68.1	28.8	6.1	***	2	2.7
*Scabiosa columbaria* L.	V1	91.7	18.8	6.7	***	2	1.4
*Thymus pulegioides* L. s. l.	V1	100.0	16.3	6.2	***	6	1.2
*Tragopogon pratensis* L	V1	46.3	18.4	5.3	***	6	0.5
*Viola declinata*	V1	45.8	20.4	5.1	***	6	0.5
*Trisetum flavescens* (L.) P. Beauv.	V2	47.3	29.6	2.2	***	6	23.0
*Alchemilla vulgaris* L.	V2	36.9	28.4	1.6	***	6	8.0
*Hieracium aurantiacum* L.	V2	30.0	13.8	5.5	*	2	0.5
*Hypericum maculatum* Crantz	V2	48.0	31.5	3.2	***	2	4.0
*Rumex acetosa* L.	V3	37.1	29.3	2.8	***	X	3.0
*Veronica chamaedrys* L.	V3	40.0	30.8	3.01	***	6	3.4
*Agrostis capillaris* L.	V4	45.5	29.9	2.32	***	4	64.6
*Taraxacum officinale* Weber ex Wiggers	V4	38.5	29.6	2.72	***	6	3.1

V—experimental variants; V1—control (no fertilization); V2—50N–25P–25K; V3—100N–50P–50K; V4—150N–75P–75K mineral fertilization treatments; INDVAL—indicative value; ADM—abundance–dominance average; Std. Dev.—standard deviation; Semnif. *** *p* ˂ 0.001; * *p* ˂ 0.05; N—nitrogen preference (Ellenberg N); X—species indifferent to trophicity/plants with high tolerance to nitrogen supply.

**Table 2:** Effect of mineral fertilization on dry matter yield (2015–2017).

Variant	Dry Matter Yield (t ha^−1^)	% Compared to Control (V1 = 100%)	Difference Compared to Control (t ha^−1^)	Significance
V1	2.79	100.0	0.00	Mt.
V2	3.67	131.9	0.89	***
V3	4.80	172.2	2.01	***
V4	5.41	194.2	2.62	***

Mt—control variant; Semnif. *** *p* < 0.001; V1—control (no fertilization); V2—50N–25P–25K; V3—100N–50P–50K; V4—150N–75P–75K mineral fertilization treatments.

**Table 3:** Differences in dry matter yield between treatments and their significance (2015–2017).

**Variant**	**Dry Matter Harvest t/ha**	**Variations in Increasing Order of Harvest**		
		**V2**	**V3**	**V4**
		**Dry Matter t/ha**		
		3.67	4.80	5.41
V1	2.79	0.89	2.01	2.62
V2	3.67		1.12	1.74
V4	4.80			0.61
V3	5.41			

V1—control (no fertilization); V2—50N–25P–25K; V3—100N–50P–50K; V4—150N–75P–75K mineral fertilization treatments.

**Table 4:** Significant difference (DS) values for treatment comparison limits (2015–2017).

The Error of Averages SX = 0.10 (t/ha)			
Distance in classification	V2	V3	V4
Values q	3.20	3.34	3.42
Theoretical DS values	0.31	0.32	0.33

V1—control (no fertilization); V2—50N–25P–25K; V3—100N–50P–50K; V4—150N–75P–75K mineral fertilization treatments.

**Table 5:** Species distribution, dry matter yield, and PCoA correlations across mineral fertilization levels.

Species	Variant (NPK kg/ha)	ADM (%)	Dry Matter Yield t/ha	Correlation with Axa 1 PCoA	Correlation with Axa 2 PCoA
*Anthoxanthum odoratum* L. s. str.	0	4.4	2.79	−0.81	0.05
*Briza media* L.	0	0.7	2.79	−0.50	0.46
*Cynosurus cristatus* L.	0	0.5	2.79	−0.82	0.04
*Festuca rubra* L.	0	19.5	2.79	−0.91	0.37
*Carex pallescens* L.	0	0.5	2.79	−0.64	0.18
*Luzula multiflora* (Ehrh.) Lej	0	0.5	2.79	−0.74	0.05
*Lotus corniculatus* L	0	1.2	2.79	−0.57	−0.10
*Trifolium pratense* L.	0	2.6	2.79	−0.49	0.12
*Trifolium repens* L.	0	3.3	2.79	−0.71	−0.09
*Carlina acaulis* L.	0	0.5	2.79	−0.63	0.55
*Cerastium glomeratum* Thuill	0	0.5	2.79	−0.53	0.49
*Gentiana lutea* L.	0	0.5	2.79	−0.54	0.47
*Gymnadenia conopsea* (L.) R. Br. s. l.	0	0.5	2.79	−0.70	0.60
*Leontodon autumnalis* L.	0	0.5	2.79	−0.74	0.09
*Leucanthemum vulgare* Lam. s. str.	0	2.3	2.79	−0.75	0.54
*Plantago lanceolata* L.	0	2.6	2.79	−0.65	0.49
*Plantago media* L.	0	3.8	2.79	−0.72	0.56
*Polygala vulgaris* L.	0	0.7	2.79	−0.54	0.36
*Potentilla erecta* (L.) Raeusch.	0	2.7	2.79	−0.66	0.32
*Scabiosa columbaria* L.	0	1.4	2.79	−0.54	0.33
*Thymus pulegioides* L. s. l.	0	1.2	2.79	−0.55	0.49
*Tragopogon pratensis* L	0	0.5	2.79	−0.81	0.03
*Viola declinata*	0	0.5	2.79	−0.88	−0.17
*Trisetum flavescens* (L.) P. Beauv.	50N25P25K	23.0	3.67	−0.06	−0.97
*Alchemilla vulgaris* L.	50N25P25K	8.0	3.67	−0.14	−0.67
*Hieracium aurantiacum* L.	50N25P25K	0.5	3.67	−0.52	−0.13
*Hypericum maculatum* Crantz	50N25P25K	4.0	3.67	−0.46	−0.50
*Rumex acetosa* L.	100N50P50K	3.0	4.8	+0.46	−0.48
*Veronica chamaedrys* L.	100N50P50K	3.4	4.8	+0.26	−0.04
*Agrostis capillaris* L.	150N75P75K	64.6	5.41	+0.98	+0.18
*Taraxacum officinale* Weber ex Wiggers	150N75P75K	3.1	5.41	+0.59	−0.09

ADM (%)—average dominant abundance of each species within the experimental treatments; PCoA Axis 1/Axis 2—Pearson correlation coefficients between species distribution and the first two axes of the principal coordinate analysis; NPK (kg ha^−1^)—mineral fertilization levels applied to each treatment (V1: 0; V2: 50N–25P–25K; V3: 100N–50P–50K; V4: 150N–75P–75K).

**Table 6:** MRPP results climatic variation in floristic composition of the control grassland.

Groups Compared	T Statistic	A (Within-Group Agreement)	*p*-Value
T1 vs. T2	4.31777002	0.44352461	0.00549029
T1 vs. T3	−4.36935279	0.43398510	0.00538084
T2 vs. T3	−3.92988266	0.23000792	0.00577857

Note. T—test statistic, A—agreement statistic; T1—year 2015; T2—year 2016; T3—year 2017.

**Table 7:** Indicator species were identified for the control variants under the climatic conditions of the study years (2015–2017).

Scientific Name of the Species	Variant	IND Value	*p* *
*Briza media* L.	1	78.1	0.0072
*Festuca rubra* L.	1	39.0	0.0072
*Trisetum flavescens* (L.) P. Beauv.	3	62.7	0.0054
*Carex pallescens* L.	3	71.4	0.0054
*Alchemilla vulgaris* L. s.l.	1	53.5	0.0072
*Campanula patula* L.	1	66.7	0.0056
*Centaurea pseudophrygia* C.A. Mey.	3	44.4	0.0054
*Pimpinella major* (L.) Huds.	2	42.7	0.0274
*Thymus pulegioides* L.	2	64.5	0.0286

Note: experimental year (1 = 2015, 2 = 2016, 3 = 2017) in which the species reached its maximum indicator value; IND Value—indicative value (%) expressing the strength of association between the species and the corresponding year; * *p*—significance level.

**Table 8:** Importance of axis.

Axis	Degree of Participation (r)	Cumulative
1	0.879	0.896
2	0.113	0.992

Note. r is the correlation coefficient between ordination distances and original distances in the n-dimensional space.

**Table 9:** Correlation of environmental and productivity variables with PCoA axes.

Variable	r (Axa 1)	r^2^	tau	r (Axa 2)	r^2^	tau	r (Axa 3)	r^2^	tau
Dry Matter Yield	−0.952	0.907	−0.727	−0.200	0.040	−0.091	0.064	0.004	0.182
TMA	0.046	0.002	−0.284	0.885	0.783	0.782	0.385	0.149	0.284
PA	−0.776	0.602	−0.284	−0.542	0.294	−0.355	−0.275	0.075	−0.284
TM_MAMI	−0.607	0.369	−0.284	0.708	0.501	0.782	0.275	0.076	0.284
P_AMI	−0.708	0.502	−0.284	−0.611	0.374	−0.355	−0.301	0.091	−0.284
TM_Mar	−0.178	0.032	−0.284	0.874	0.764	0.782	0.369	0.136	0.284
TM_Apr	−0.832	0.692	−0.284	−0.472	0.222	−0.355	−0.247	0.061	−0.284
TM_Mai	0.656	0.430	0.284	0.657	0.431	0.355	0.318	0.101	0.284
TM_Iun	−0.937	0.879	−0.284	−0.277	0.077	−0.355	−0.168	0.028	−0.284
P_Mar	0.054	0.003	0.284	−0.886	0.784	−0.782	−0.381	0.145	−0.284
P_Apr	0.054	0.003	0.284	−0.886	0.784	−0.782	−0.381	0.145	−0.284
P_Mai	−0.615	0.378	−0.284	0.703	0.494	0.782	0.273	0.074	0.284
P_Iun	−0.491	0.241	−0.284	−0.764	0.583	−0.355	−0.356	0.127	−0.284

Note: TMA—annual mean temperature (°C); PA—annual precipitation amount (mm); TM_MAMI—mean temperature of March–June (°C), representing the main growth period; P_AMI—precipitation amount of March–June (mm), corresponding to the main growth period; TM_Mar/TM_Apr/TM_Mai/TM_Iun—mean monthly temperatures for March, April, May, and June (°C); P_Mar/P_Apr/P_Mai/P_Iun—monthly precipitation amounts for March–June (mm).

**Table 10:** The monthly average temperatures recorded at Gheţari weather station (2015–2017).

Year	Months												Average
	I	II	III	IV	V	VI	VII	VIII	IX	X	XI	XII	
2015	−0.8	2	4.7	3.8	10.8	13.5	16.5	16.5	12.7	7.7	3.4	−0.2	7.7
2016	−5.4	0.1	1	8	8.7	14.8	16	15.3	10.9	4.6	0.4	−5.4	5.7
2017	−1.8	6	8.3	6.4	10	14.6	15.5	15.3	10.7	7.9	0.6	−3.2	7.5
**Average values for the period 2001–** **2017**													
2001–2017	−4.5	−2.7	0.2	5.3	10.5	14.5	15.9	15.4	11.3	6.0	1.4	−3.1	5.8

**Table 11:** The monthly average precipitations recorded at Gheţari weather station (2015–2017).

Year	Months												Total
	I	II	III	IV	V	VI	VII	VIII	IX	X	XI	XII	
2015	32.6	14.2	23.6	48.6	69.4	78.2	33.8	95	124	38.8	98.4	49.8	706.4
2016	120	111.4	79.4	108.4	67	165.4	58.8	49.4	56	86.6	127	0.2	1030
2017	112	0	86	11.4	116.6	95	37	35	88	100	98	36.2	815.2
**Average values for the period 2001–2017**													
2001–2017	67.2	55.9	81.8	77.2	102.4	100.3	137.3	98.4	92.6	86.6	86.5	55.8	1042.1

**Table 12:** Modified Braun–Blanquét scale for assessing the abundance–dominance of plant species, based on classes and sub-classes (after [112]).

Class	Coverage Interval (%)	Class Central Value (%)	Sub-Note	Sub-Interval (%)	Central-Adjusted Value of Sub-Interval (%)
5	75–100	87.5	5c	92–100	96
			5b	83–92	87.5
			5a	75–83	79
4	50–75	62.5	4c	67–75	71
			4b	58–67	62.5
			4a	50–58	54
3	25–50	37.5	3c	42–50	46
			3b	33–42	37.5
			3a	25–33	29
2	10–25	17.5	2c	20–25	22.25
			2b	15–20	17.5
			2a	10–15	12.5
1	1–10	5	1c	6–10	8
			1b	4–6	5
			1a	1–4	2.5
+	0.1–1	0.5	-	-	0.5
r	0.01–0.1	0.05	-	-	0.05

Note: a, b, c indicate the sub-note of each class.

## Data Availability

The original contributions presented in this study are included in the article. Further inquiries can be directed to the corresponding author.
